# OP26 Judicialization Of High-Priced Technologies In Uruguay: Evolution, Approach To Strategies And Their Impact

**DOI:** 10.1017/S0266462325100846

**Published:** 2025-12-29

**Authors:** Alejandra Croci, Alejandra Ferrari, Gervasio Cabrera, Magdalena Irisarri, Javier Pintos, Ana Deminco, Alicia Alemán, Ana Pérez Galán

## Abstract

**Introduction:**

Judicialization processes are a tool used by the population to access benefits not included in health coverage. New technologies present challenges in terms of financing health systems. Uruguay has suffered a substantial increase in the last decade in writs of protection and health costs associated with them. Different actions have been taken in order to reduce judicialization throughout these years.

**Methods:**

We performed a descriptive study of the evolution of the number of writs of protection in Uruguay in the period 2012 to 2023. The data were obtained by analyzing requests for access to public information available on the web, requested by different stakeholders. Types of technologies and their frequency were analyzed. The costs per year were obtained from the national government’s accounts. The strategies implemented in each period were obtained from regulations available on the website of the Ministry of Health (MoH).

**Results:**

Writs of protection have substantially increased, with a total of 124 in 2012 and increasing to 1,750 in 2023. The MoH’s spending on writs of protection rose from two percent in 2012 to 59 percent of its overall spending in 2023. Different measures taken by the MoH in the period 2014 to 2017 were introduced with the intention of reducing judicialization; examples include advice to lawyers from experts in health technology assessment, abbreviated administrative processes, and interinstitutional committees for price negotiation. Also, coverage of the most demanded technologies was a strategy used in most recent years.

**Conclusions:**

In Uruguay, writs of protection continue to increase constantly. The different strategies implemented have not achieved a real impact in terms of quantity and costs associated with writs of protection. The recent creation of the Health Technology Agency may contribute to reducing the impact of judicialization on the health system.

